# Epidemiology of salivary gland tumours in an Eastern Caribbean nation: A retrospective study

**DOI:** 10.1016/j.amsu.2018.10.039

**Published:** 2018-11-08

**Authors:** Leon Noel, Steve Medford, Shariful Islam, Alyssa Muddeen, Wesley Greaves, Solaiman Juman

**Affiliations:** a Department of Otorhinolaryngology and Head and Neck Surgery, San Fernando Teaching Hospital, Trinidad and Tobago; b Department of General Surgery, San Fernando Teaching Hospital, Trinidad and Tobago; c Department of Pathology, San Fernando Teaching Hospital, Trinidad and Tobago; d Department of Clinical Surgical Science, University of the West Indies, St. Augustine, Trinidad and Tobago; e Department of Otorhinolaryngology and Head and Neck Surgery, Eric William Medical Science Complex, Trinidad and Tobago

**Keywords:** Parotid, Submandibular, Sublingual and salivary gland

## Abstract

**Objective:**

The epidemiology of the salivary glands tumour is not well documented in the Caribbean countries. Therefore, the aim of this study is to determine the local trend of salivary gland tumours with a review of current diagnostic techniques.

**Design & Methods:**

Retrospective data was collected from the electronic database at the Pathology department of the San Fernando Teaching Hospital between the periods January 2005 to June 2015. All patients who underwent primary resection of either the parotid, submandibular or minor salivary glands for diagnosed tumour cytologically or suspected tumour were included in this study. The clinical and histopathological data were then collected and analyzed.

**Results:**

A total of 85 surgeries were performed for suspected or diagnosed neoplasia, 54 parotidectomies, 26 submandibular gland and 5 minor salivary gland excisions. The benign neoplastic lesions, pleomorphic adenoma and Warthin's tumour, were the most common 53 (62.4%) of all the resections performed, followed by non-neoplastic lesions 25 (29.4%) such as sialadenitis, cysts or normal glands. Malignant neoplasms made up the minority with only 7 cases whereby mucoepidermoid carcinoma was the most common malignant neoplasm found followed by squamous cell carcinoma.

**Conclusion:**

Parotid gland remains the most frequent site of salivary gland tumours (80%), with pleomorphic adenoma being the most common benign tumour. Triple assessment is still required to manage these cases adequately with stress on preoperative tissue diagnosis FNAB vs USS guided core biopsy.

## Highlights

• Salivary gland tumours are very rare of all head and neck tumours.
• Parotid gland remains the most frequent site of salivary gland tumours (80%).
• Pleomorphic adenoma is the most common benign tumour of the parotid gland with a slight male predominance.
• Squamous cell carcinoma was the most common malignant neoplasm of the parotid gland in contrast to worldwide data in which muco-epidermoid and adenoid cystic carcinomas are the most common.
• Triple assessment is required to manage these cases adequately with stress on preoperative tissue diagnosis by FNAC or USS guided core biopsy.

## 1 Introduction

The epidemiology of salivary gland tumours is poorly documented in our region. In international series they are an uncommon entity comprising 3–4% of all head and neck neoplasms with varying sites of origin, histopathology and clinical findings. Commonly involved are the parotid, submandibular and minor salivary glands of the palate, but the sublingual gland is rarely affected [1,2]. Anatomically the parotid gland is the most frequent site of the salivary gland tumours (80–85%), where three quarter (75%) of the lesions are benign and 25% are malignant [2,3]. Less frequently, salivary gland tumours originate in the submandibular, sublingual and minor salivary glands, which are located near the mandible and throughout the submucosa of the oral cavity and upper aerodigestive tract respectively. In contrast to tumours originating from the parotid gland, 40–45% of the submandibular gland tumours, 70–90% of the sublingual gland tumours and 50–75% of minor salivary glands are malignant [3].

The most common benign tumour is pleomorphic adenoma, followed by Warthin's tumour. The most common malignant salivary gland tumours are mucoepidermoid carcinoma (34%) followed by adenoid cystic carcinoma (22%) and adenocarcinoma (18%) [2].

Various studies looking at the epidemiology and histopathological subtypes of salivary gland neoplasms have been conducted in the USA, Europe, Asia and South America. However, there is very little data available about the incidence, demographics and histopathological distribution in our country or in the Caribbean. The aim of this study is to investigate the local trends of salivary gland tumours in Trinidad and Tobago. The data from this study should help us understand the various presentations, epidemiology and review our current diagnostic techniques and management.

## 2 Design and method

A ten year retrospective review was conducted at the San Fernando Teaching Hospital (SFTH) between the period January 2005 to June 2015. Data was collected from the electronic database of the Department of Pathology using the keywords “parotid”, “submandibular”, “Sublingual” and “salivary gland”. All tumours resected at our institute during this time period were included. The patients selected all underwent parotidectomy, submandibular gland, sublingual gland or minor salivary gland resection and the specimens were reviewed by the senior pathologist. A total of 108 cases were performed. Twenty three cases were excluded as the medical files could not be retrieved. The demographics – age, gender, comorbidities and histopathological data were reviewed and analyzed. The work has been reported in line with the STROCSS criteria [4].

## 3 Results

**Fig. 1:**
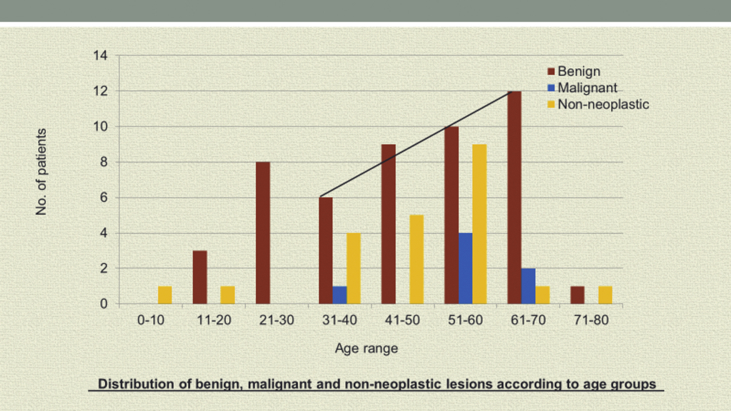
Distribution of benign, malignant and non-neoplastic lesions according to age groups.

A study sample of 85 resections were reviewed and analyzed. The mean age of the patients reviewed was 46.8 years with a range 0f 6–72 years showing a slight male preponderance of 1: 1.2. Benign neoplastic lesions were the most common 53 (62.4%) of all the resections performed, followed by non-neoplastic lesions 25 (29.4%) such as sialadenitis, cysts or normal glands. Malignant neoplasms made up the minority with only 7 cases. There were no documented sublingual gland lesions. Fig. 1 shows the age range for benign, malignant and non-neoplastic disease in which the age range 51–60 years had the highest incidence of all lesions.

### 3.1 Parotid gland lesions

The parotid gland was the most common site of the tumours resected accounting for 54 (63.5%) of all resections. Pleomorphic adenoma was found to be the most common benign neoplasm in the parotid gland 29 followed by Warthin's tumor 10. The four (4) malignant neoplasms removed were an acinic cell carcinoma, a mucoepidermoid carcinoma, a salivary duct carcinoma and a squamous cell carcinoma each.

### 3.2 Submandibular gland lesions

Twenty six (26) submandibular gland (SMG) resections were performed the majority being for non-neoplastic lesions, 13 sialoadenitis and 4 normal gland due to previous sialolithiasis. There were 8 benign neoplasm (7 pleomorphic adenomas and 1 Warthin's tumour). There was 1(one) metastatic basaloid squamous cell carcinoma from the tongue. There was equal number of male and female patients who had resections of the SMG.

### 3.3 Minor salivary gland lesions

Five (5) minor salivary gland excisions were documented, three (60%) were benign while two (40%) were malignant. The two malignant tumours were mucoepidermoid carcinomas, both being females. Table 1 shows a summary of the distribution of the various histological subtypes.

**Table 1:** Distribution of various histological subtypes according to location and gender.

	PAROTID	SUBMANDIBULAR	MINOR	FEMALE	MALE	TOTAL
Pleomorphic Adenoma	29	7	2	21	17	38
Warthin's tumor	10	1	0	0	11	11
Basal Cell Adenoma	2	0	1	2	1	3
Mucoepidermoid CA	1	0	2	2	1	3
Acinic Cell CA	1	1	0	0	1	1
Squamous Cell CA	1	0	0	0	2	2
Salivary Duct Carcinoma	1	0	0	0	1	1
Oncocytoma	1	0	0	0	1	1
Total	46	9	5	25	35	60

Only twenty-five FNAB were conducted preoperatively and it showed a sensitivity and specificity of 71% and 100% respectively with a false negative rate of 29%.There were no changes made to any previous diagnosis after review by the pathologist.

## 4 Discussion

Trinidad and Tobago is a twin island in the eastern Caribbean region with an estimated population of 1.3 million. In Trinidad and Tobago little information is available on the clinical presentation and histopathology of salivary gland tumours.

In our setting we found mucoepidermoid carcinoma comprising 43% while squamous cell carcinoma followed comprising 29% of all malignancies. Of note, majority of the mucoepidermoid carcinomas were excised from minor salivary glands whereas all the squamous cell carcinomas were found in the parotid gland and submandibular gland respectively.

Locally, Ramdass et al. [5] conducted a study on 60 cases of parotid gland tumours in the other 3 general hospitals in Trinidad and Tobago. The majority of the benign lesions (73.2%) were similarly found to be pleomorphic adenomas and Warthin's tumor however the primary malignant lesions accounted for (26.8%) with squamous cell carcinoma being the most common.

Regionally, A study from Jamaica by Williams et al. [6] analyzing data of 446 salivary gland biopsies 99 (21.3%) were non-neoplastic and 365 (78.7%) were neoplasms: 261 (71.5%) benign and 104 (28.5%) malignant. Similarly pleomorphic adenoma was the most common benign tumour and mucoepidermoid carcinoma was found to be the most common malignant lesion. This findings are consistance with our study as well as with others recent studies [[7], [8], [9]].

We found that most patients presented with indolent masses which gradually increased in size over the years. None of the patients presented with facial weakness, cervical lymphadenopathy or distant metastasis to suggest malignancy. In contrary to other studies there were more males who presented with salivary gland disease (1:1.2). As for most masses these patients underwent triple assessment: clinical examination, radiological imaging and pathology.

Most common radiologic investigation utilized in our setting was ultrasonography because it has the advantage of being inexpensive, non-invasive, simple to perform and can differentiate cystic from solid lesions [10]. It may also enhance the accuracy of FNAB in nonpalpable tumours and in masses with highly heterogeneous architecture [11]. Computerized tomography was reserved for causes suspected involving the deep lobe of parotid gland or other structures. Magnetic resonant imaging (MRI) scans are also a great imaging modality in the workup of these patients; however it is expensive and not readily available in our setting.

Generally the accuracy is higher for benign than for malignant salivary gland tumours [12]. Sharma and associates [13] demonstrated that the impact of FNAB altered decision making and prevented the need for surgery in 40% of cases. Heller [14] also showed that by surgeons knowing the diagnosis preoperatively this resulted in a change in clinical approach in 35% of patients. This meant avoiding surgical resections for lymphomas and inflammatory masses and conservative approaches for benign lesions and high risk surgical patients. Ultrasound-guided core biopsy offers potential advantages over FNAB that include both improved consistency and diagnostic accuracy. However, safety profiles relative to tumor seeding, capsule rupture, and hematoma still need to be clearly established [15,16].

The limitation of our study is that it is a retrospective study and the size of the sample is small. The authors have decided to conduct a future study to include all of these patients from all of the hospital of the country.

## 5 Conclusion

In our study, we compared local epidemiological data to regional and international data with respect to histopathological classification. Males were affected more with pleomorphic adenoma and mucoepidermoid carcinoma being the most common lesions of the salivary glands. Triple assessment is still required to manage these cases adequately with stress on preoperative tissue diagnosis FNAB vs USS guided core biopsy.

## Consent

Patients consent is not needed as it is a retrospective audit. However, approval was obtained from institutional review board to conduct this study.

## Ethical approval

It is a retrospective study. However, approval was obtained from institutional review board to conduct this study.

## Author contribution

Leon Noel , Alyssa Muddeen, Steve Medford and Shariful Islam are involved in conceptualization, data curation; writing original draft and formal analysis of the draft. Wesley Muddeen and Solaiman Juman are involved in formal analysis and writing - review & editing the draft.

## Sources of funding

No funding of was received to publish this study.

## Conflicts of interest

The authors have nothing to disclose.

## Trial registry number

Research Registry entry- UIN - 4355.

## Guarantor

All authors have accepted full responsibility for the work and all have agreed to publish the manuscript.

## Provenance and peer review

Not commissioned, externally peer reviewed.

## Appendix A Supplementary data

the_strocss_statement_checklist the_strocss_statement_checklist
